# High-performance hybrid modeling chemical reactors using differential evolution based fuzzy inference system

**DOI:** 10.1038/s41598-020-78277-3

**Published:** 2020-12-04

**Authors:** Meisam Babanezhad, Iman Behroyan, Ali Taghvaie Nakhjiri, Azam Marjani, Mashallah Rezakazemi, Saeed Shirazian

**Affiliations:** 1 Institute of Research and Development, Duy Tan University, Da Nang, 550000 Vietnam; 2 Faculty of Electrical–Electronic Engineering, Duy Tan University, Da Nang, 550000 Vietnam; 3 Faculty of Mechanical and Energy Engineering, Shahid Beheshti University, Tehran, Iran; 4 Department of Petroleum and Chemical Engineering, Science and Research Branch, Islamic Azad University, Tehran, Iran; 5 Department for Management of Science and Technology Development, Ton Duc Thang University, Ho Chi Minh City, Vietnam; 6 Faculty of Applied Sciences, Ton Duc Thang University, Ho Chi Minh City, Vietnam; 7 Faculty of Chemical and Materials Engineering, Shahrood University of Technology, Shahrood, Iran; 8 Laboratory of Computational Modeling of Drugs, South Ural State University, 76 Lenin prospekt, 454080 Chelyabinsk, Russia

**Keywords:** Mathematics and computing, Computational science, Mechanical engineering

## Abstract

Bubbly flow behavior simulation in two-phase chemical reactors such bubble column type reactors is widely employed for chemical industry purposes. The computational fluid dynamics (CFD) approach has been employed by engineers and researchers for modeling these types of chemical reactors. In spite of the CFD robustness for simulating transport phenomena and chemical reactions in these reactors, this approach has been known as expensive for modeling such turbulent complex flows. Artificial intelligence (AI) algorithm of the adaptive network-based fuzzy inference system (ANFIS) are largely understood and utilized for the CFD approach optimization. In this hybrid approach, the CFD findings are learned by AI algorithms like ANFIS to save computational time and expenses. Once the pattern of the CFD results have been captured by the AI model, this hybrid model can be then used for process simulation and optimization. As such, there is no need for further simulations of new conditions. The objective of this paper is to obviate the need for expensive CFD computations for two-phase flows in chemical reactors via coupling CFD data to an AI algorithm, i.e., differential evolution based fuzzy inference system (DEFIS). To do so, air velocity as the output and the values of the x, and y coordinates, water velocity, and time step as the inputs are inputted the AI model for learning the flow pattern. The effects of cross over as the DE parameter and also the number of inputs on the best intelligence are investigated. Indeed, DEFIS correlates the air velocity to the nodes coordinates, time, and liquid velocity and then after the CFD modeling could be replaced with the simple correlation. For the first time, a comparison is made between the ANFIS and the DEFIS performances in terms of the prediction capability of the gas (air) velocity. The results released that both ANFIS and DEFIS could accurately predict the CFD pattern. The prediction times of both methods were obtained to be equal. However, the learning time of the DEFIS was fourfold of ANFIS.

## Introduction

Different types of chemical reactors are being employed in chemical and biochemical engineering industries such as plug flow and batch reactors. However, continuous chemical reactors have attracted much attention due to better process efficiency. Among the various reactors applicable of process industry, bubble column types are mainly used in different disciplines, e.g., water and wastewater treatment, separation/purification, and biopharmaceuticals manufacturing. These reactors are continuous type and provide higher mass transfer rates, and lower operational and fixed costs^1–4^. These efficient reactors have been well understood by experimental and theoretical works in which the effect of underlying parameters on the reactor performance have been studied by underpinning work^5–12^. The results turned out that it is of great importance to well comprehend the turbulent behavior of these reactors.

The basic principles for optimizing and scaling up of the bubble columns is usually built by the experimental analysis (e.g., DoE), empirical/semi-empirical correlations, population balance model (PBM), and uni/multi-dimensional convection–dispersion models. Notwithstanding, the aforementioned approaches remain limited in simple cases. Regarding this issue, 3D CFD (computational fluid dynamics) computational techniques are of beneficial to be employed for understanding the bubble column reactors and unlock the process complexity. Indeed, in this context, CFD contributes to comprehending the complex turbulent flow with two phases within the reactor considering the interactions between two phases. This versatile and novel computational method has considerably attracted attention in the past decades for simulation of complex fluid systems mainly for design, optimization, understanding, and process troubleshooting purposes^5^.

CFD models have been used for a multidimensional bubble column taking into account an Eulerian approach^13^. In another research, Bhusra and co-workers^5,6^ employed CFD technique for modeling, simulation, and understanding air–water interaction in a chemical reactor, in which Eulerian technique was assumed for modeling and simulation purposes. A bubble column was modelled in another study^14^ with employing *VOF* technique as the CFD method. Chen et al.^15^ investigated gas hold-up distribution, turbulency behavior of fluids, and velocity of liquid. An adiabatic bubbly flow model with two fluids was implemented^16^. Circulation of liquid phase and combining in the column with internals and without them were studied^17^.

Thermal/hydrodynamic behavior of gas/liquid flow inside the bubble column rectors have been investigated experimentally by Kalaga and co-workers^7–9^ where they studied hydrodynamics in the bubble column via the methods of experimental particle tracking. In spite of the robustness of these methods for understanding the flow pattern in bubble column reactors, the aforementioned techniques are disable for finding the relations that exist among such characteristics. In this context, Artificial Intelligence (AI) science can be employed and tailored for the bubble column reactors for better understanding and developing these chemical reactors. Fuzzy inference system (FIS) which is an intelligence engine can be employed in this context for process simulation and optimization^18–20^. Linking FIS and adaptive network (AN) was employed and reported in a few studies^21,22^, but no investigations exist for considering the other AI algorithms.

In order to address the mentioned research gap in the field of chemical reactors, in the present paper, a bubble column reactor considering air–water as fluids is simulated and the results of the CFD computations are learned by an appropriate AI method called *DEFIS*. The main idea of using *DEFIS* is that to develop a correlation between the velocities of air and water in each time and position (e.g., x y z) inside the column. For the first time, a comparison is made between the ANFIS and the DEFIS performances in terms of the prediction of air speed.

## Methodology

### Reactor shape and structure

The shape and design of the reactor for the computational task in this work is a reactor in cylindrical shape (diameter = 290 mm, length = 2 m). The reactor is equipped with a nozzle having the diameter of 23 cm. The air is injected into the reactor, initially filled with water as liquid phase with the temperature and velocity of 22 ºC and 5 mm/s, respectively.

### CFD method

For the CFD simulations in this work, two-fluid Euler-Euler approach was employed with appropriate CFD code. The main equations can be expressed as^23,24^:

- Continuity equation:
  1 $${\frac{\partial }{{\partial t}}}\left( {{\in _{k}}{ \rho_{k}}}\right) + {\frac{\partial }{{\partial x_{i}}}}({\in _{k}}{\rho_{k}} {u_{{k,i}}}) = 0$$
- Momentum equation:
  2 $$\frac{\partial }{\partial t}\left({\in }_{k}{\rho }_{k}{u}_{k,i}\right)+{u}_{j}\frac{\partial }{\partial {x}_{j}}\left({\in }_{k}{\rho }_{k}{u}_{k,i}\right)=-{\in }_{k}\frac{\partial P}{\partial {x}_{i}}+{\in }_{k}{\rho }_{k}g+\frac{\partial }{\partial {x}_{j}}\left({\in }_{k}\left(\mu +{\mu }_{t}\right)\frac{\partial {u}_{k,i}}{\partial {x}_{j}}\right)+{F}_{I}$$

The energy conservation equation was employed to estimate the heat transfer between air and water inside the reactor, and $k-\varepsilon$ turbulence model was used for the turbulency^5,25^.

### The differential evolution (DE) algorithm

To discover x for optimizing $f (\text{x})$; x = [× 1, × 2, × 3, …, xD] is the common problem in an optimization algorithm. *D* represents the function dimensionality. The variables’ domains are denoted by their upper and lower bounds: ${x}_{j,upp}, {x}_{j,low}; j\in \{1,\dots ,D\}$*. NP D*-dimensional vectors are included in the original DE algorithm population^26–28^:

3 $${{x}_{i,G}=\{x}_{i},1,G,{x}_{i},2,G,\dots ,{x}_{i},D,G\}, \quad i=\text{1,2},\dots ,NP,$$

*G* represents the generation. Crossover and mutation operations are used by DE for producing a trial vector:

4 $${{u}_{i,G}=\{u}_{i},1,G,{u}_{i},2,G,\dots ,{u}_{i},D,G\}, \quad i=\text{1,2},\dots ,NP,$$

#### Mutation operation

A mutant vector is created by mutation for every population vector as:

5 $${{x}_{i,G}\Rightarrow {v}_{i,G}=\{v}_{i},1,G,{v}_{i},2,G,\dots ,{v}_{i},D,G\}, \quad i=\text{1,2},\dots ,NP,$$

One of the mutation approaches can be used to create the mutant vector. The most convenient approaches include^29^:

- ‘rand/1’:
  6 $${v}_{i,G}={x}_{{r}_{1},G}+F \cdot \left({x}_{{r}_{2},G}-{x}_{{r}_{3},G}\right)$$
- ‘best/1’:
  7 $${v}_{i,G}={x}_{best,G}+F \cdot \left({x}_{{r}_{1},G}-{x}_{{r}_{2},G}\right)$$
- ‘current to best/1’:
  8 $${v}_{i,G}={x}_{i,G}+F \cdot \left({x}_{best,G}-{x}_{i,G}\right)+F \cdot \left({x}_{{r}_{1},G}-{x}_{{r}_{2},G}\right)$$
- ‘best/2’:
  9 $${v}_{i,G}={x}_{best,G}+F \cdot \left({x}_{{r}_{1},G}-{x}_{{r}_{2},G}\right)+F \cdot \left({x}_{{r}_{3},G}-{x}_{{r}_{4},G}\right)$$
- ‘rand/2’:
  10 $${v}_{i,G}={x}_{{r}_{1},G}+F \cdot \left({x}_{{r}_{2},G}-{x}_{{r}_{3},G}\right)+F \cdot \left({x}_{{r}_{4},G}-{x}_{{r}_{5},G}\right)$$

In Eqs. (6–10) the indices ${r}_{1}$, ${r}_{2}$, ${r}_{3}$, ${r}_{4}$, ${r}_{5}$ stand for the various and random mutually integers created in the range of $[1, NP]$ that are different also from index *i*. A mutation scale factor is represented by *F* in the range of [0, 2] (< 1). ${x}_{best,G}$ represents the best vectors within generation *G*.

#### Crossover procedure

The crossover process can be expressed using Eq. (11):

11 $$\begin{aligned} u_{{i,j.g}} = \left\{ {\begin{array}{*{20}l} {v_{{i,j.g}} ,} \hfill & {if\;rand({\text{0,1}}) \le CR\;or\;j = j_{{rand}} ,} \hfill \\ {x_{{i,j.g}} ,} \hfill & {otherwise} \hfill \\ \end{array} } \right. \hfill \\ i = {\text{1,2}}, \ldots ,NP\;and\;j = {\text{1,2}}, \ldots ,D \hfill \\ \end{aligned}$$

The crossover parameters or factors are within the [0, 1] denoting the probability of generating the trial vector parameters from a mutant vector^26–28^.

### Fuzzy inference system (FIS)

The artificial intelligence approach known as FIS which is an efficienct calculating framework was used here for linking with the CFD framework. In the FIS structure, a fuzzy reasoning was employed on the basis of if–then rules^30^. Herein the x, and y coordinates, water velocity (V_w_), and time are considered to attain air velocity in the reactor as the model’s output in the predictions using AI. The AI rule for the i^th^ level is found as^30^:

12 $${w}_{i}=K\left(X\right) L\left(Y\right)M({V}_{w})N(t)$$

where ${w}_{i}$ denotes the signal exiting the node in layer 2. Also, *K, L, M, N* denote the signals incoming from MFs. Details of FIS are reported elsewhere^23,31^.

## Results and discussion

**Figure 1:**
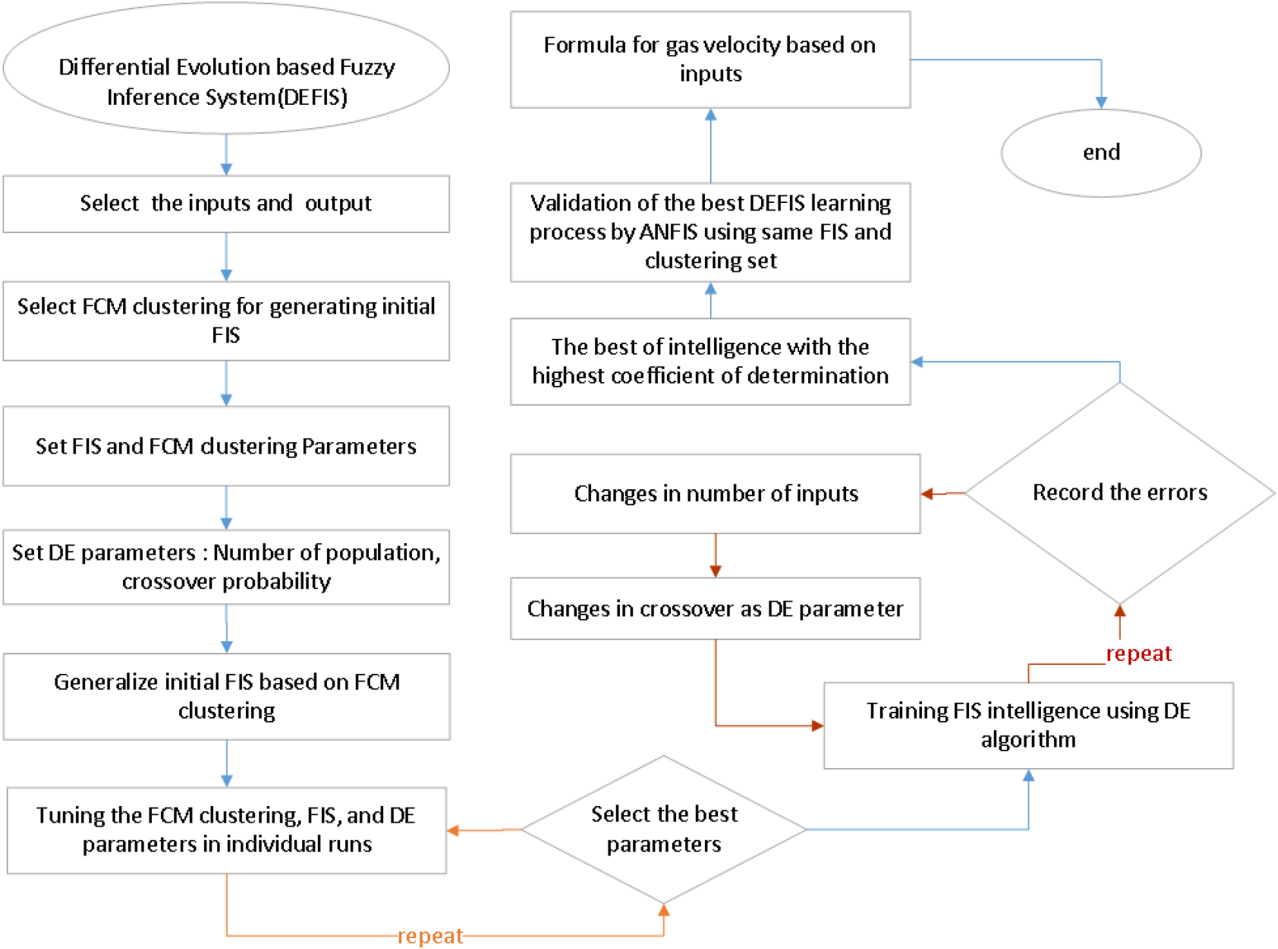
Flowchart of differential evolution based fuzzy inference system (DEFIS).

Figure 1 describes the DEFIS setup and validation as a flowchart for the prediction of the air speed inside the co-current reactor based on the x, and y coordinates, water velocity (V_w_), and time. Once the inputs (x, and y coordinates, water velocity, and time) and the output (air velocity) have been determined, the fuzzy C-means clustering (FCM) is employed for generating inertia FIS. Then the FCM and the FIS variables must be defined accordingly. In terms of the differential evolution (DE) parameters, the number of population and crossover probability is determined. In order to achieve the best intelligence, a sensitivity analysis was carried out for different input numbers and crossover. The accuracy of the DEFIS in the prediction of the air velocity is checked with the CFD data, and the coefficient of determination (R^2^) is recorded in the computations. Once the highest value of R^2^ has been obtained, the sensitivity analysis must be stopped. Further validation is done by repeating the same process using the ANFIS. The results of DFIS are compared with those of ANFIS. Finally, a correlation is developed for calculation of the air velocity inside the co-current bubble column reactor as a function of the x, and y coordinates, water velocity (V_w_), and time. The AI algorithms could cooperate with the CFD for finding such relationships. So, the AI algorithm of the DEFIS has been used in combination with the CFD modeling. Two-phase flow of air–water inside a co-current bubble column reactor is simulated using Eulerian CFD model at different time steps (e.g. 10, 20, 30, 40, 50 s). The air velocity is the output variable of the DEFIS. The x, and y coordinates, water velocity (V_w_), and time are the input data learned by the DEFIS.

**Figure 2:**
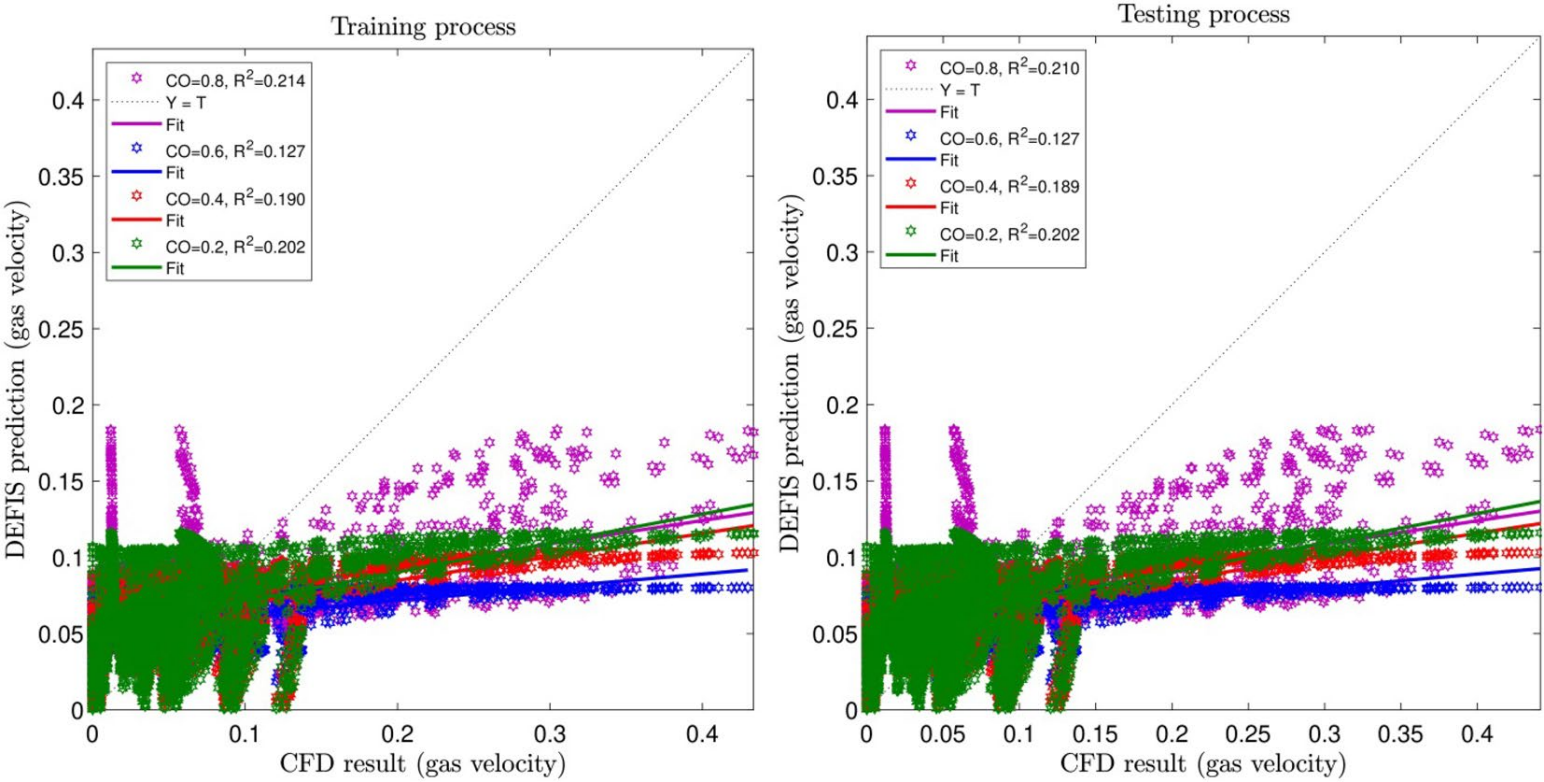
Learning processes of DEFIS method with two inputs and changes in crossover (CO).

**Figure 3:**
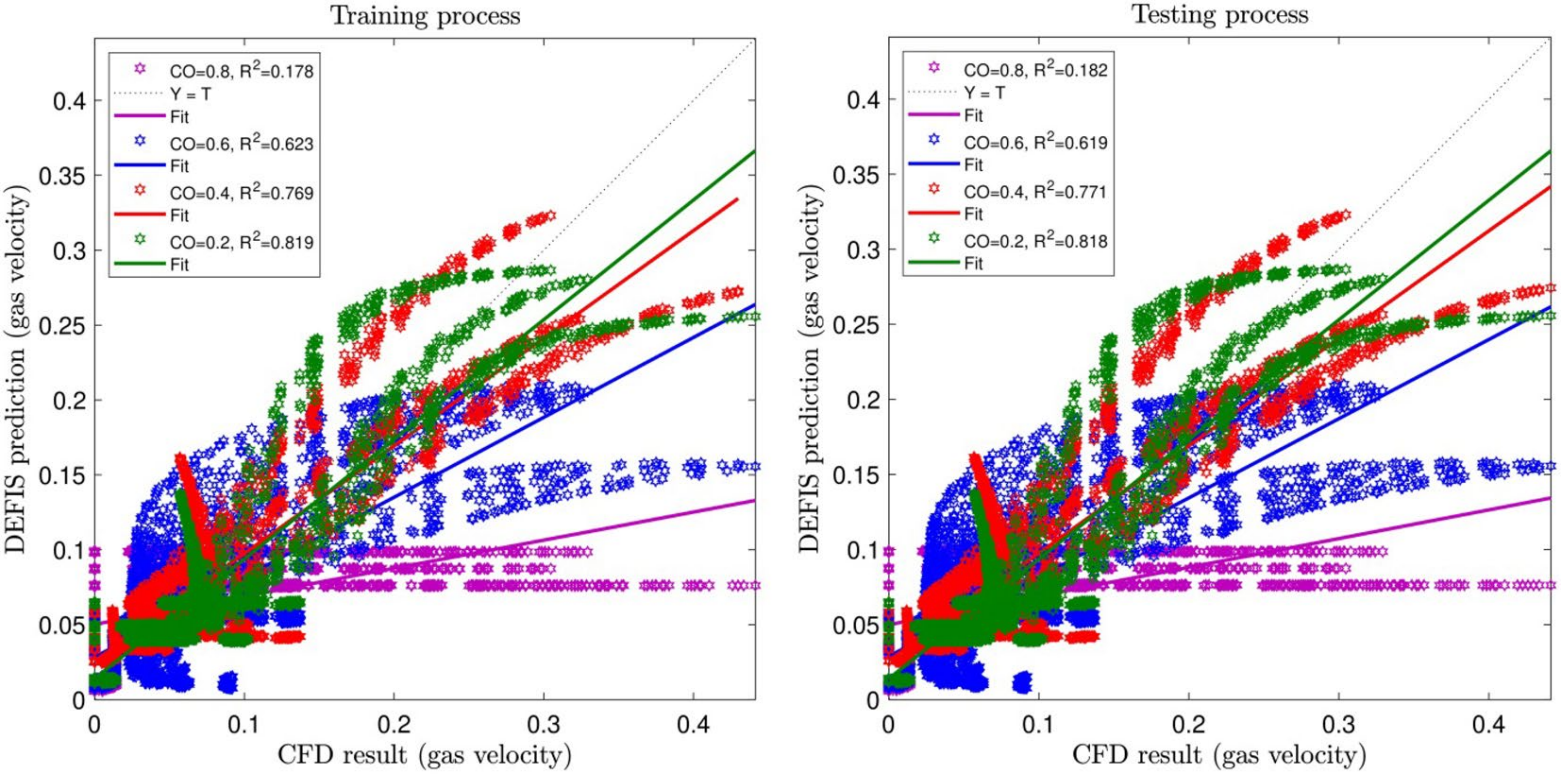
Learning processes of DEFIS method with three inputs and changes in crossover (CO).

**Figure 4:**
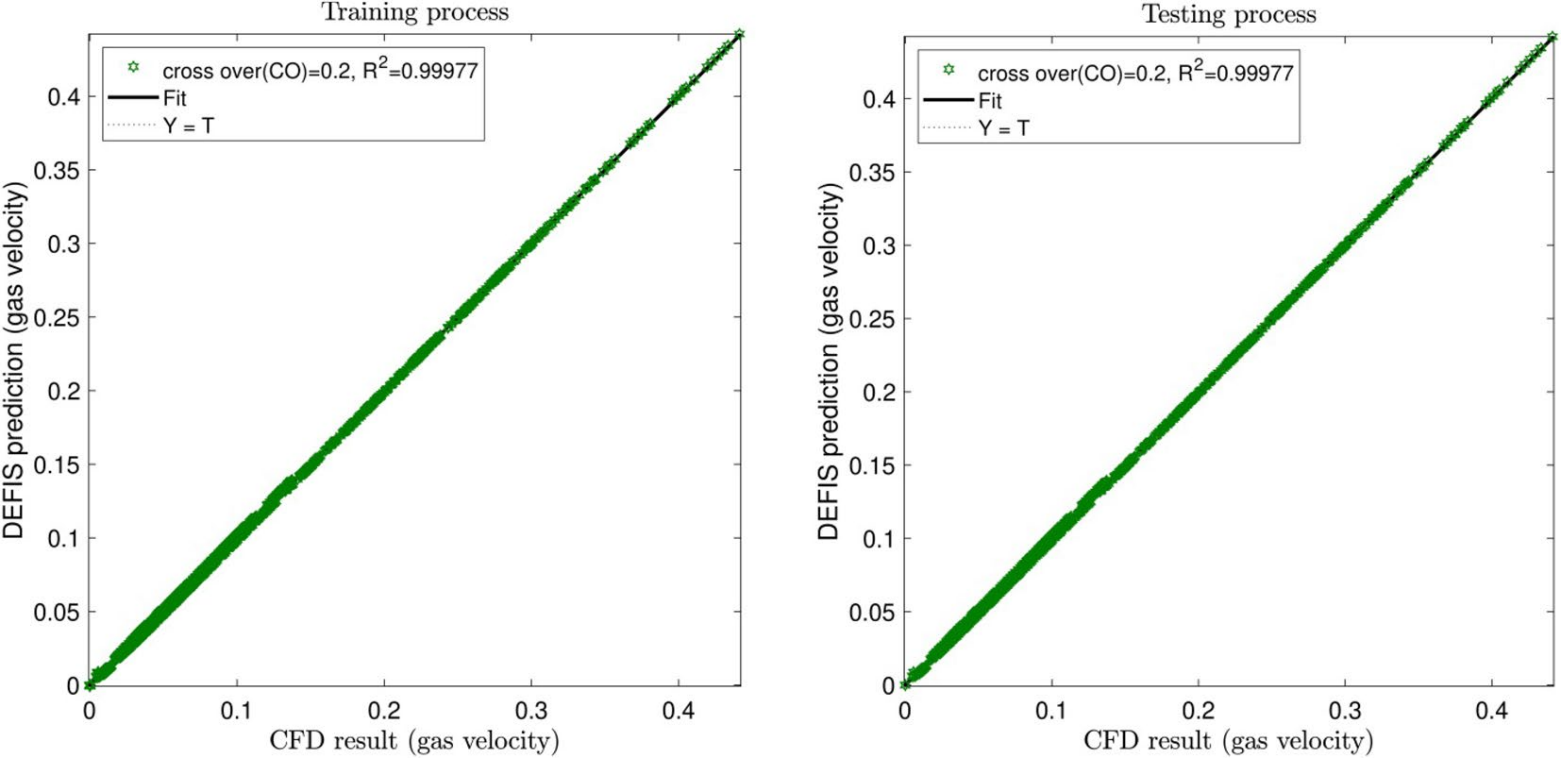
Learning processes of DEFIS method with four inputs and crossover (CO) = 0.2.

In this study, 75% of the CFD data are used in training AI algorithm. But the whole data are used in the testing approach. Figures 2, 3 and 4 illustrate the changes of the regression number (R^2^) by adjusting the input number and the cross-over (CO) parameter, respectively. As can be seen, the highest R^2^ is obtained for the case when 4 inputs are considered in the model. However, it is not always the case, increasing the input numbers, the R^2^ increases. Sometimes, especially when there are not logical relations between the selected inputs and the output, adding more inputs the regression number falls down, and this might impair the calculations. Regarding CO, the highest R^2^ (i.e. 0.99977) is for 0.2. Hence, the best intelligence condition is seen for the input numbers of 4 and CO equal to 0.2.

**Figure 5:**
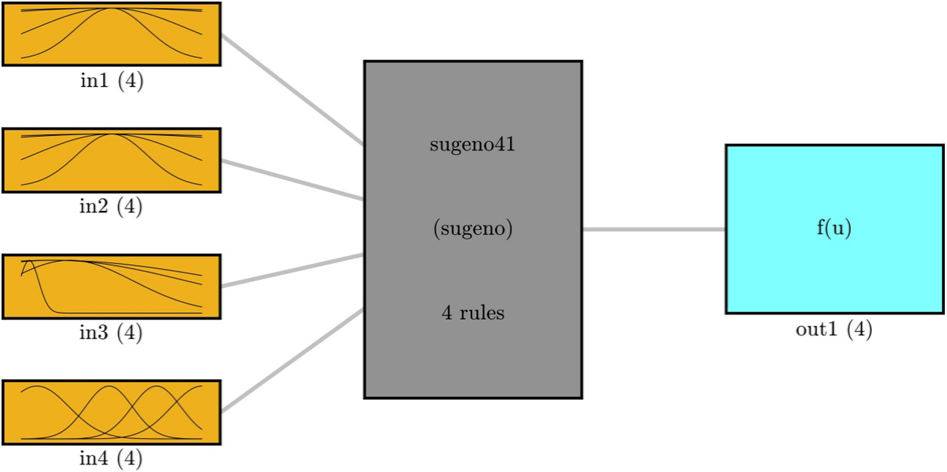
DEFIS structure in the high level of intelligence.

Figure 5 depicts the FIS structure being used in this paper for the process simulations. The type of cluster is semi-fuzzy clustering, and 4 clusters are used for each input. The number of rules and output memberships are also set to 4. Gaussian is the type of membership function that is shown schematically in the rectangular boxes on the right-hand side.

The Gaussian function is illustrated in Table 1. According to the function, *c* and *σ* are the inputs membership functions parameters calculated by the DEFIS learning process in each cluster. Table 2 shows the values of *c* and *σ* of each cluster at the best intelligence condition.

**Table 1:** Mathematical formula of Gaussian function used in this work.

Membership function	Formula
Gaussian	${e}^{\frac{-{\left(x-c\right)}^{2}}{2{\sigma }^{2}}}$

**Table 2:** The parameters of input membership functions in the highest intelligence of DEFIS method.

	Number of cluster	Type of MFs	σ	c
First input	'in1cluster1'	'gaussmf'	− 5.2257E−02	4.2513E−04
	'in1cluster2'	'gaussmf'	5.8232E−01	3.2302E−04
	'in1cluster3'	'gaussmf'	− 1.2828E+01	− 1.7492E−03
	'in1cluster4'	'gaussmf'	− 1.2653E−01	5.2875E−02
Second input	'in2cluster1'	'gaussmf'	5.0518E−02	− 1.1089E−03
	'in2cluster2'	'gaussmf'	1.4579E−01	− 6.1457E−04
	'in2cluster3'	'gaussmf'	2.6418E−01	5.6296E−04
	'in2cluster4'	'gaussmf'	2.5247E−01	− 8.6988E−04
Third input	'in3cluster1'	'gaussmf'	2.8153E−01	7.7039E−02
	'in3cluster2'	'gaussmf'	7.3711E+00	1.1823E−02
	'in3cluster3'	'gaussmf'	2.2778E−01	1.6959E−02
	'in3cluster4'	'gaussmf'	3.7091E−01	− 2.8461E−01
Forth input	'in4cluster1'	'gaussmf'	6.6080E+00	3.9870E+01
	'in4cluster2'	'gaussmf'	1.3802E+00	3.1087E+02
	'in4cluster3'	'gaussmf'	5.4194E+00	1.1682E+01
	'in4cluster4'	'gaussmf'	− 9.1207E+00	2.8001E+01

**Figure 6:**
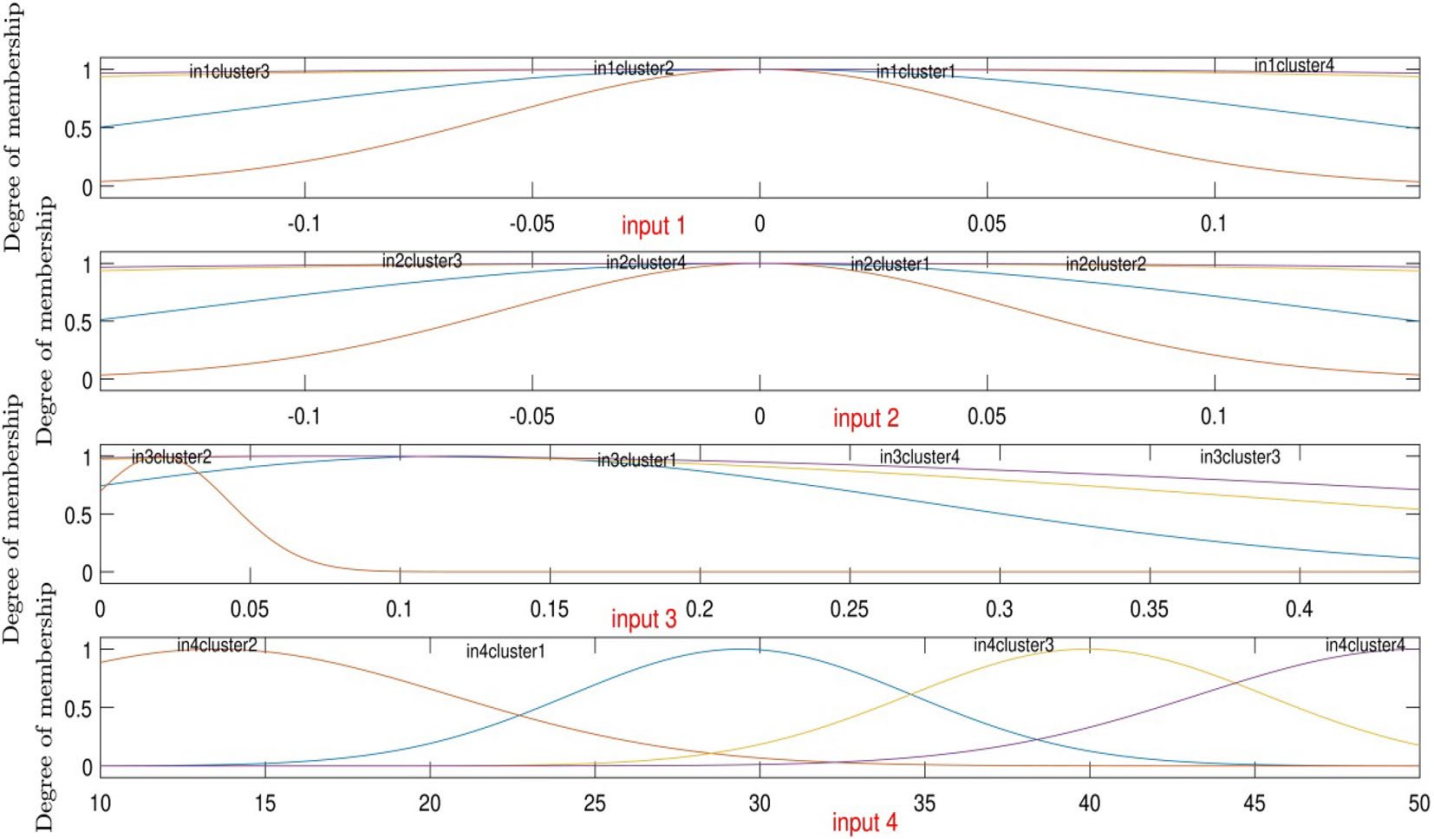
Inputs membership in the high level of intelligence.

Gaussian function changes versus the changes of inputs are shown in Fig. 6. According to this figure, the value of Gaussian function (µ) for each input and cluster can be determined. As mentioned before, consequent parameters for predicting air velocity is also obtained after reaching the best intelligence.

**Figure 7:**
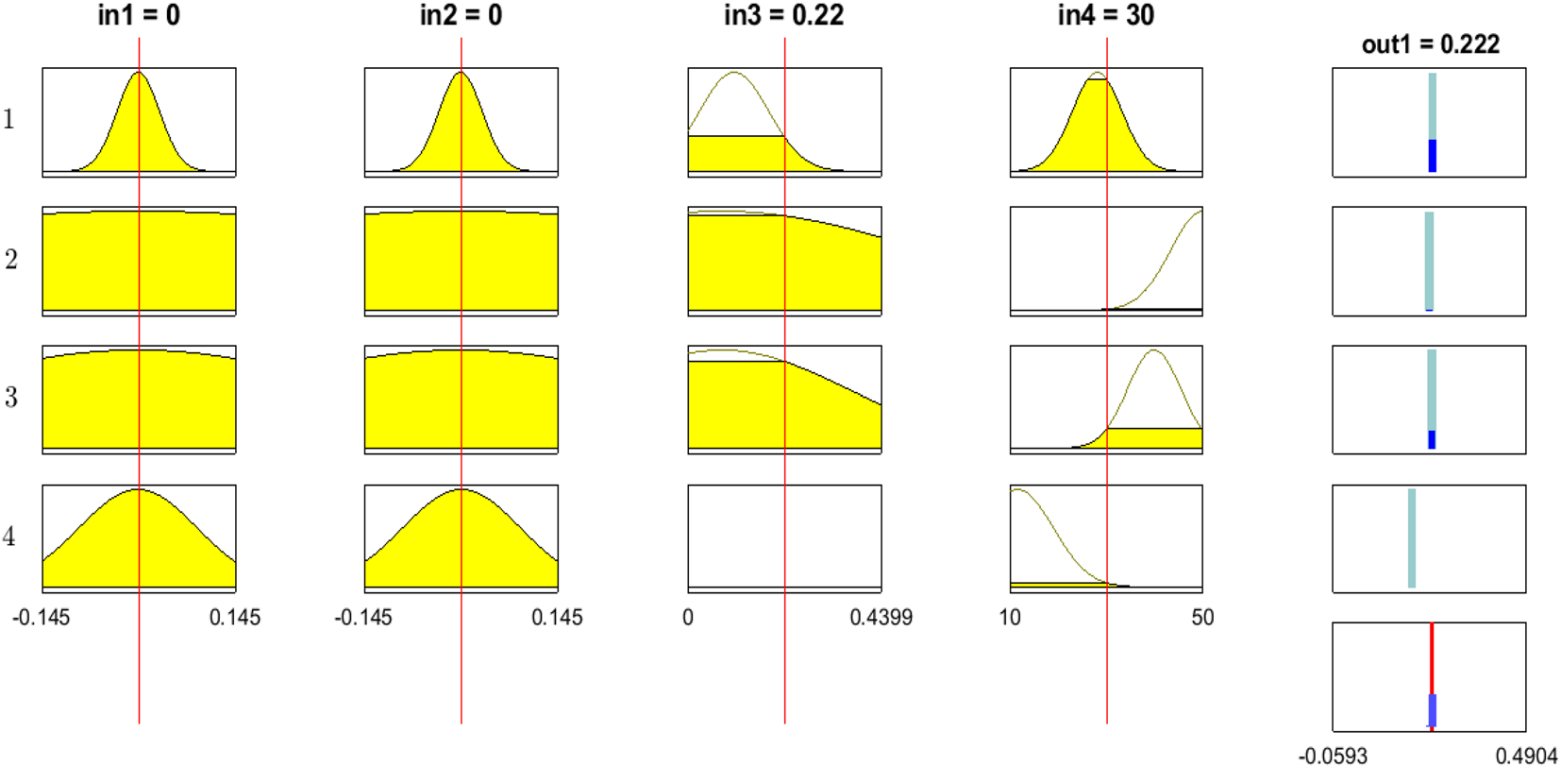
Fuzzy reasoning procedure of prediction of air velocity inside the reactor by using the trained rules and trained membership functions.

Figure 7 illustrates the fuzzy reasoning procedure of prediction of air velocity inside the BCR. The operations of fuzzy reasoning are based on comparisons of the input parameters with the MFs on the premise part and obtaining the values of membership for each linguistic label^32^. Table 3 shows these values for every cluster. So, using Eq. (13), the formula is obtained to correlate the air velocity to x, y, time, and V_w_.

**Table 3:** DEFIS method consequent parameters for predicting gas velocity in z direction.

Output MFs	Output MFs type	o	p	q	r	s
'out1cluster1'	'linear'	2.6131E−04	2.3510E−05	1.0059E+00	− 1.2047E−05	6.3183E−04
'out1cluster2'	'linear'	− 3.0254E−04	− 6.6650E−04	1.3127E+00	− 7.0574E−05	− 1.5252E−04
'out1cluster3'	'linear'	2.1228E−04	2.9970E−04	1.0058E+00	− 1.5727E−05	4.0862E−04
'out1cluster4'	'linear'	2.3020E−04	− 4.5140E−04	1.0059E+00	− 3.5953E−05	1.9991E−04

As a summary, once the best intelligence of the DEFIS is achieved, all parameters of the Gaussian membership function and the consequent parameters could be found. Replacing the parameters in Eq. (13), the air velocity could be calculated as a function of x, y, time, and V_w_. In this way, the air velocity is calculated in the domain using the formula without additional CFD modeling. This means that there is no need to discretize and solve the complicated governing equations (mass, momentum, etc.) by the CFD tool anymore. As a result, the computational efforts are facilitated by the artificial intelligence of the DEFIS. In other words, the DEFIS helps the users to save the computational time and costs by the elimination of the CFD modeling for prediction of the air velocity in new conditions (the new time and/or the new water velocity).

13 $${V}_{g}=\frac{\sum_{i=1}^{4}\sum_{j=1}^{4}\sum_{k=1}^{4}\sum_{l=1}^{4}\left({\mu }_{1i}\times {\mu }_{2j}\times {\mu }_{3k}\times {\mu }_{4l}\right)\times \left({o}_{m}X\times {p}_{m}Y\times {q}_{m}{V}_{l}\times {r}_{m}Time\times {s}_{m}\right)}{\sum_{i=1}^{4}\sum_{j=1}^{4}\sum_{k=1}^{4}\sum_{l=1}^{4}\left({\mu }_{1i}\times {\mu }_{2j}\times {\mu }_{3k}\times {\mu }_{4l}\times {\mu }_{5n}\right)}$$

**Figure 8:**
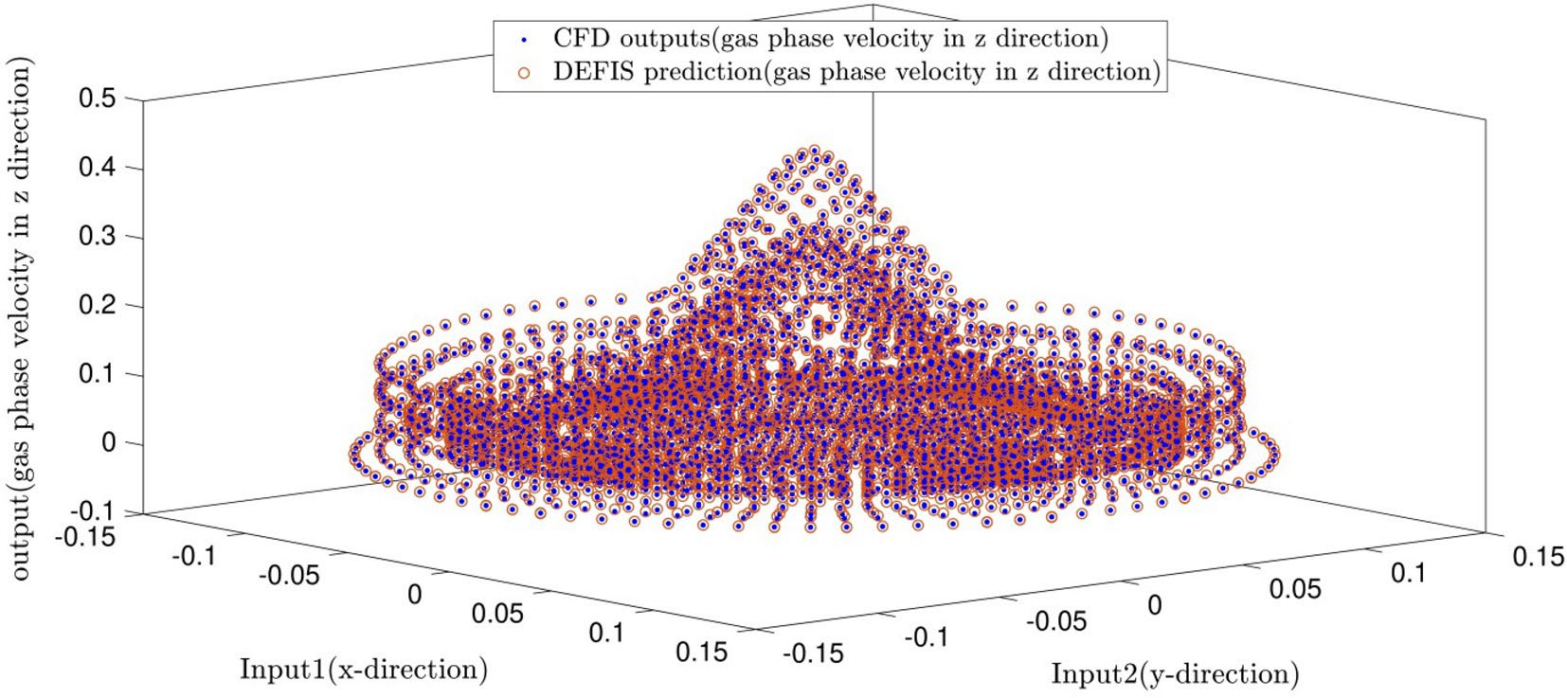
DEFIS prediction and its comparison with CFD results.

To further assess the model developed in this work, comparisons between the CFD computations in terms of air speed and the DEFIS predictions have been determined and illustrated in Fig. 8. The air has been injected into the bottom side of the reactor vessel along the axial direction. This figure illustrates the axial air velocity in a cross-section plane of the cylindrical column placing in height of 10 cm from the bottom. The blue points represent the air velocities of the nodes predicted by the CFD, while the red ones show those predicted by the DEFIS. The results reveal that both methods predict the same air speeds with minimum deviations.

For more validations and comparisons, the CFD computations data are learned by the other AI method of ANFIS once again. According to Table 4, the similar setup parameters have been chosen for both ANFIS and DEFIS. At this condition, although the prediction time of both methods is equal, the learning time of DEFIS is fourfold of ANFIS.

**Table 4:** Learning and prediction times for the similar setup parameters of DEFIS and ANFIS.

Method	DEFIS	ANFIS
Number of inputs	4	4
Maximum of iteration	800	800
Percentage of data in training processes	75%	75%
Percentage of data in testing processes	100%	100%
Clustering type	Fuzzy C-mean Clustering	Fuzzy C-mean Clustering
Type of input membership function	Gaussmf	Gaussmf
FIS type	Sugeno	Sugeno
Number of cluster for each input as FCM clustering parameter	4	4
Number of rules	4	4
Exponent as FCM clustering parameter	2	2
Minimum improvement as FCM clustering parameter	1.00E−05	1.00E−05
Learning time(s)	172.3169051	43.8744104
Prediction time(s)	0.086412	0.0884874

**Figure 9:**
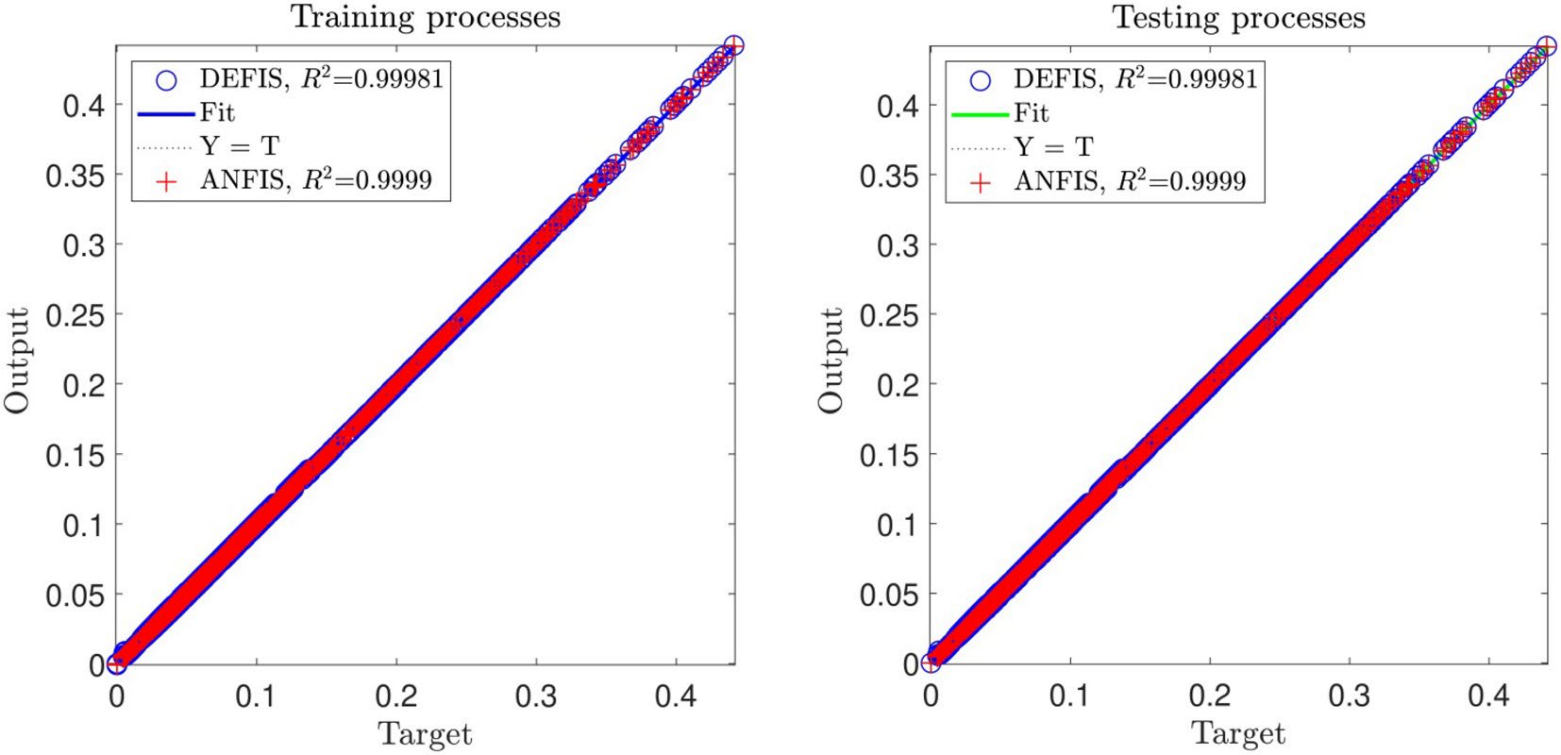
Validation of DEFIS method by comparison with ANFIS method.

**Figure 10:**
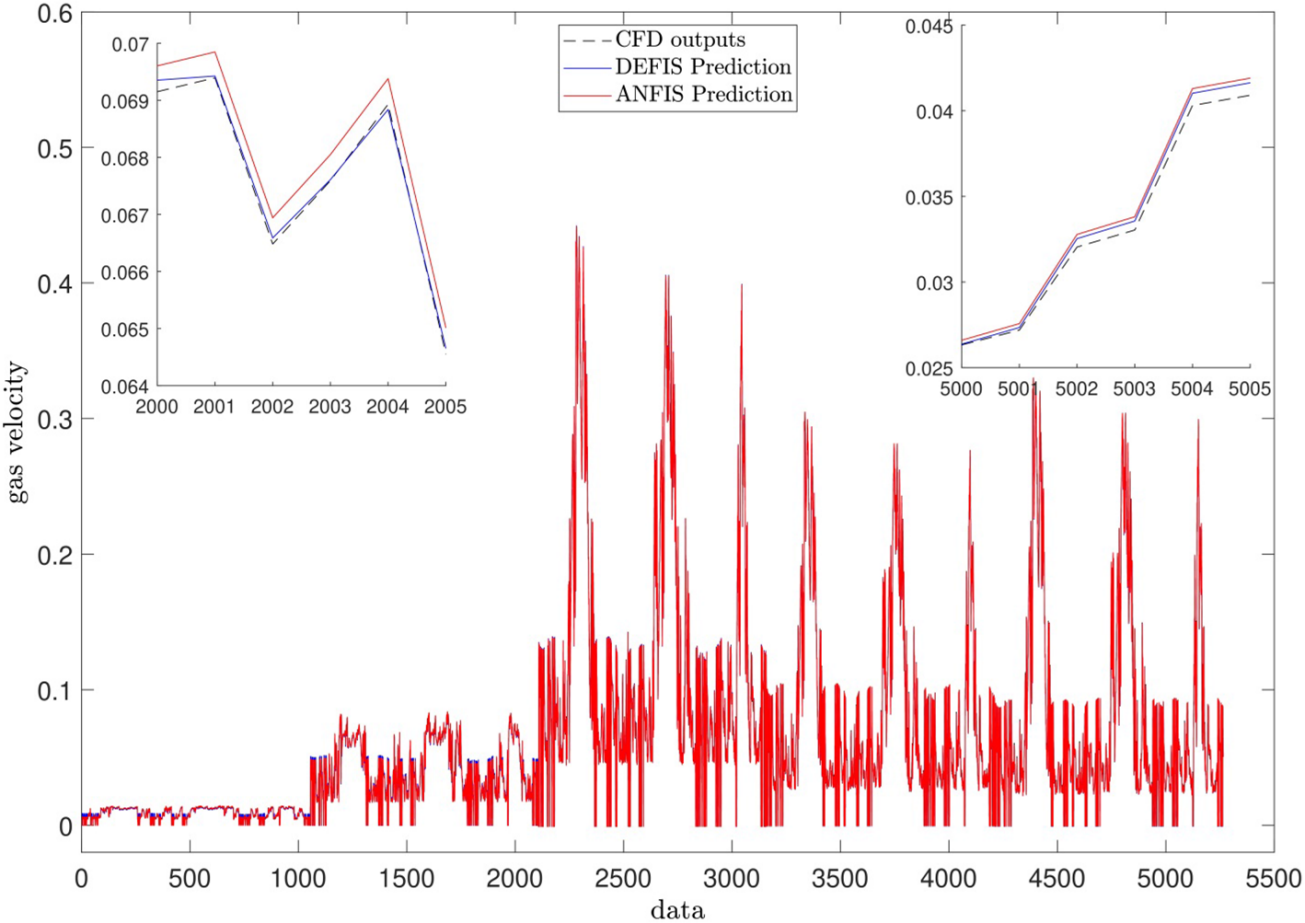
Performance comparison between DEFIS and ANFIS in the prediction of the CFD results.

For accuracy in prediction, as shown in Fig. 9, the coefficient of determination (R^2^) is close to 1 in both methods at the best intelligence. This similar performance in the prediction of the CFD results (the air velocity) is also shown in Fig. 10. Totally, 5265 CFD data have been used in this study for the computations. As seen in Fig. 10, the CFD results are closely predicted by both ANFIS and DEFIS for the whole of 5265 data.

## Conclusion

CFD modeling of an air–water co-current bubble column reactor was considered in the present study. The artificial intelligence (AI) came to help the CFD model by learning the generated data. After learning and capturing the general pattern of the CFD results by the AI algorithms, there is no need for further simulations of new conditions by expensive computational tasks. The AI algorithm of the differential evolution based fuzzy inference system was selected, as the main method for learning the CFD data. For further validation, a comparison was made between the DEFIS and the ANFIS performances in the prediction of the air velocity. The Eulerian two-phase CFD model was employed to predict air velocity inside the reactor at different time steps. The x, and y coordinates, water velocity (V_w_), and time were considered as the input data learned by the DEFIS. The cross over as the DE parameter and also the number of inputs were adjusted for the best intelligence. Then the Gaussian membership function parameters (i.e. *c* and *σ*) and the consequent parameters were obtained by the DEFIS for all 4 clusters to correlate the air velocity based on the x, and y coordinates, water velocity (V_w_), and time. This formula eliminates needing further CFD modeling for air speed prediction. The best intelligence condition (i.e. R^2^ = 0.99977) was seen for the input numbers of 4 and CO equal to 0.2. A comparison between the CFD predictions of the air velocity and the DEFIS predictions revealed that both methods predicted the same air velocities. Comparing ANFIS and DEFIS, both methods could accurately predict the CFD results. The prediction times of both methods were equal. However, the learning time of the DEFIS was fourfold of the ANFIS.
